# Recent Advances in Exploration and Biotechnological Production of Bioactive Compounds in Three Cyanobacterial Genera: *Nostoc, Lyngbya*, and *Microcystis*

**DOI:** 10.3389/fchem.2019.00604

**Published:** 2019-09-03

**Authors:** Nguyen Huy Thuan, Tran Tuan An, Anil Shrestha, Nguyen Xuan Canh, Jae Kyung Sohng, Dipesh Dhakal

**Affiliations:** ^1^Center for Molecular Biology, Institute of Research and Development, Duy Tan University, Danang, Vietnam; ^2^Department of Life Science and Biochemical Engineering, Sun Moon University, Chungnam, South Korea; ^3^Faculty of Biotechnology, Vietnam National University of Agriculture, Gialam, Hanoi, Vietnam; ^4^Department of BT-Convergent Pharmaceutical Engineering, Sun Moon University, Chungnam, South Korea

**Keywords:** *Cyanobacteria*, *Nostoc*, *Lyngbya*, *Microcystis*, bioactive compounds, multi-omics approaches

## Abstract

*Cyanobacteria*, are only Gram-negative bacteria with the capacity of oxygenic photosynthesis, so termed as “Cyanophyta” or “blue-green algae.” Their habitat is ubiquitous, which includes the diverse environments, such as soil, water, rock and other organisms (symbiosis, commensalism, or parasitism, etc.,). They are characterized as prominent producers of numerous types of important compounds with anti-microbial, anti-viral, anti-inflammatory and anti-tumor properties. Among the various cyanobacterial genera, members belonging to genera *Nostoc, Lyngbya*, and *Microcystis* possess greater attention. The major reason for that is the strains belonging to these genera produce the compounds with diverse activities/structures, including compounds in preclinical and/or clinical trials (cryptophycin and curacin), or the compounds retaining unique activities such as protease inhibitor (micropeptins and aeruginosins). Most of these compounds were tested for their efficacy and mechanism of action(MOA) through *in vitro* and/or *in vivo* studies. Recently, the advances in culture techniques of these cyanobacteria, and isolation, purification, and chromatographic analysis of their compounds have revealed insurmountable novel bioactive compounds from these cyanobacteria. This review provides comprehensive update on the origin, isolation and purification methods, chemical structures and biological activities of the major compounds from *Nostoc, Lyngbya*, and *Microcystis*. In addition, multi-omics approaches and biotechnological production of compounds from selected cyanobacterial genera have been discussed.

## Introduction

*Cyanobacteria* (green-blue algae) exist in diverse communities *viz*. terrestrial (soil, rock, sand, etc.,), marine, fresh and brackish water environments. They can also exist as symbiont, commensal or parasites with other animals/plants. They can survive in large varieties of environmental conditions, including high and low ranges of temperatures, light intensities, pH and salinity (Martinez-Frances and Escudero-Oñate, 2018). Moreover, they are only group of Gram negative prokaryotes with capacity of oxygenic photosynthesis, and possessing the chlorophyll *a*. Hence, they can grow and multiply at the simple expense of light, H_2_O, CO_2_, and inorganic nutrients (Russo and Cesario, 2012; Moreira et al., 2013). Evolutionarily cyanobacteria are considered as one of the oldest bacteria and pioneer to conquer the ancient environments (Hamilton et al., 2016; Lee, 2018). Moreover, they have diversity in morphology and physiology including the ability for production of numerous compounds, which is facilitated by their discrete primary and secondary metabolism (Cardellina and Moore, 2010). However, some compounds produced by marine cyanobacteria are toxic to human and animals, and thus also termed as “cyanotoxins” (Carmichael, 1992). For instance, microcystin and nodularin produced by brackish water cyanobacterial *Nodularia spumigena* have been reported to be hepatotoxic and carcinogenic to animal and human (Chen et al., 2013). Using the advanced techniques of separation and purification, many cyanobacterial compounds were tested, and established as new pharmaceutical leads during drug discovery and development. Subsequently they were evaluated for their biological activities *in vivo* and *in vitro* and detailed studies of their MOA were conducted vigorously (Welker et al., 2012).

A comprehensive report of cyanobacterial and algal metabolites from the point of view of basic chemicals and applications have been described (Singh et al., 2017; Martinez-Frances and Escudero-Oñate, 2018) and the detailed investigation about their chemical diversity and biological activities (anti-bacterial, anti-fungal and anti-mycobacterial activities) have been discussed (Shishido et al., 2015; Swain et al., 2017). Furthermore, the biosynthetic diversity and comparative genomics of cyanobacterial secondary metabolites have been focused (Dittmann et al., 2015; Leao et al., 2017).

Among the numerous “genera” of cyanobacteria capable to produce important secondary metabolites, the members of the genera *Nostoc, Lyngbya*, and *Microcystis* are most prominent producers of bioactive compounds with diverse activities/structures. These bioactive compounds also include compounds in preclinical and/or clinical trials (cryptophycin and curacin), or compounds with unique activities such as protease inhibitor (micropeptins). Hence, most frequently the strain belonging to these three genera were screened, and used for culture and discovery of compounds with interesting chemical diversities. Generally the interesting chemical structures are associated with promising bioactivities such as anti-fungal, anti-viral, anti-bacterial, anti-oxidant, immunosuppressive, anti-proliferative activity, etc., hence these compounds were screened for versatile bioactivity assays. These bioactivity assays, not only established the members of genera *Nostoc, Lyngbya*, and *Microcystis* as major source of bioactive compounds, but also provided information regarding specific bioactivities of the target compounds, and their mechanism of actions were studied in details (Itoh et al., 2014b; Iwasaki et al., 2015b; Luo et al., 2019). Hence, members of genera *Nostoc, Lyngbya*, and *Microcystis* are most influential candidates for exploring the bioactive compounds from marine environment.

Most of the bioactive compounds from these cyanobacteria belong to polyketides and peptides, whereas pharmaceutically important compounds belonging to terpenes, alkaloids, lipids, etc., are also reported. The polyketide compounds are bio-synthesized by enzyme complexes called as polyketide synthase (PKS), which are responsible for sequential decarboxylatic condensation of acyl-CoA precursors. The PKS belong to three major categories as Type I, II, and III (Shen, 2003; Mishra et al., 2019). The majority of peptide compounds are biosynthesized by either enzymatic complexes as non-ribosomal peptide synthase (NRPS) or as ribosomally synthesized and post-translationally modified peptides (RiPPs) (Sussmuth and Mainz, 2017; Hudson and Mitchell, 2018). However, there are many bioactive compounds which share hybrid chemical structures containing polyketide and peptide parts. Thus, the natural combination of biosynthetic enzymes belonging to PKS, NRPS, and RiPPs are responsible for defining the chemical structures and thus determining the bioactivities of these compounds derived from the members of cyanobacteria. In this paper, several properties of secondary metabolites isolated and characterized from three important genera aforementioned have been reviewed regarding their origin, isolation and purification methods, chemical structures, and significant biological activities. Furthermore, multi-omics approaches and biotechnological production of those valuable bioactive compounds is discussed.

## Chemical Analysis and Biological Activities of Several Important Compounds From *Nostoc, Lyngbya, and Microcystis*

### Nostoc

*Nostoc* are morphologically arranged in colonies that consists of filaments containing moniliform cells within a gelatinous sheath. They are routinely isolated from different environmental locations as soil, moist rocks, bottom of lakes and springs (both fresh- and saltwater), but rarely exist in marine habitats. Some members of *Nostoc* are characterized to possess a significant role in the nitrogen fixation. Besides, a significant number of *Nostoc* species are associated with production of interesting chemical structures with biological activities (Lee, 2018).

### Carbamidocyclophane

#### Origins

Carbamidocyclophane A-E and H-L were isolated from the Vietnamese *Nostoc* sp. CAVN 10 and CAVN 2, respectively (Bui et al., 2007; Preisitsch et al., 2015). Similarly, carbamidocyclophane F-G were obtained from the freshwater cyanobacterium *Nostoc* sp. UIC 10274 (Luo et al., 2014). In addition, carbamidocyclophane M-U were also isolated from *Nostoc* sp. strain CAVN2 (Preisitsch et al., 2016).

#### Isolation, Purification, and Detection Methods

Different techniques were used for purification of target compounds. For instance, carbamidocyclophanes A-E were characterized from the extract of *Nostoc* sp. CAVN 10 by bioassay-guided separation and preparative reverse phase-high pressure liquid chromatography (RP-HPLC) (Bui et al., 2007). However, carbamidocyclophanes F-G were purified and identified by multi-step approach including Diaion™ vacuum liquid chromatography (VLC), RP-HPLC, electron spray ionization-time of flight-mass spectrometry (ESI-TOF-MS) and proton-nuclear magnetic resonance (^1^H NMR) spectroscopy (Luo et al., 2014). The derivatives as carbamidocyclophanes H-L were isolated using solid phase extraction (SPE) fraction and semi-preparative RP-HPLC (Preisitsch et al., 2015). Moreover, bromo-analogs were derived by addition of potassium bromide (KBr) to the culture of *Nostoc* sp. CAVN2. The biphasic solvent system was utilized for the extraction and enrichment of bromo-carbamidocyclophanes. Similarly, chlorinated carbamidocyclophane analogs (carbamidocyclophane M-U) were successfully isolated by semi-preparative HPLC (Preisitsch et al., 2016).

#### General Chemical Structures

Structurally, all carbamidocyclophanes are characterized by the presence of [7.7] paracyclophane ring, however there is variation in their characteristic carbamido substituents in the 1,14-positions. The carbamidocyclophanes A-D contained variations in attachment of numbers of chlorine group at the end of their butyl side chains. Carbamidocyclophane E is only congenomer that does not contain chlorine in its completely symmetrical molecular structure (Bui et al., 2007).

The carbamidocyclophanes F and G contain halogen at the [7.7] paracyclophane ring with branched methyls at C-2/15. They contain chlorination at C-11/24 similar to carbamidocyclophanes A-E. Carbamidocyclophanes A-E contain carbamate groups at both C-1/14, but carbamidocyclophanes F and G have single carbamate attached at C-14 (Luo et al., 2014).

**Figure 1:**
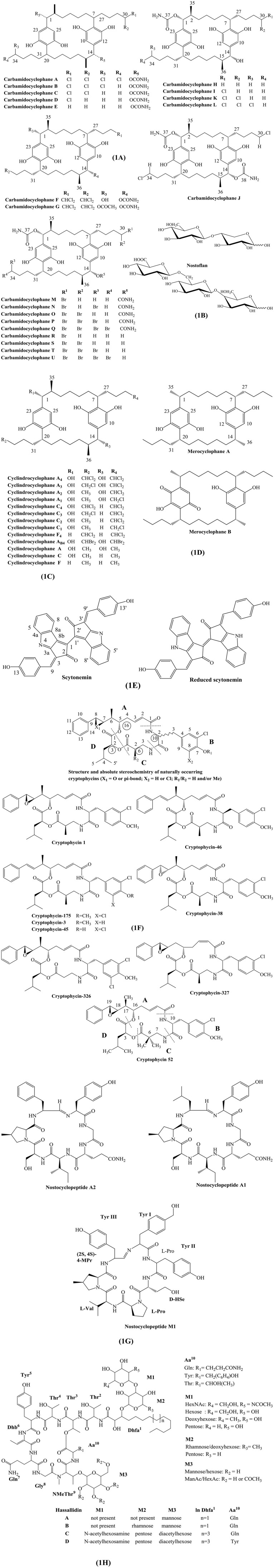
Chemical structures of bioactive molecules from *Nostoc*. **(A)** Carbamidocyclophanes, **(B)** Nostoflans, **(C)** Cyclindrocyclophanes, **(D)** Merocyclophanes, **(E)** Scytonemins, **(F)** Cryptophycins, **(G)** Nostocyclopeptides, **(H)** Hassallidins.

Preisitsch et al. (2015) isolated 5 new mono carbamoylated [7.7] paracyclophanes (H, I, K, and L), whereas each structures vary by the degree of chlorinations in their side chains. Carbamidocyclophane J contains a single halogen in both butyl residues, which is the first report of such chemical structure (Preisitsch et al., 2015). Preisitsch et al. (2016) also reported carbamidocyclophane M-U, which are brominated analogs of chlorinated carbamidocyclophanes congeners. Preisitsch et al. (2015) reported that in non-halogenated merocyclophanes, C-1/C-14 retain α-branched methyls, but C-2/C-15 lack β-branched methyl groups, respectively. Structurally, nostocyclophanes lack branched methyls, but contain chlorine atoms at C-3 and C-16, which also exist in glycosylated derivatives, such as nostocyclophane A and B. The carbamidocyclophane subgroup contain characteristics carbamoyl moieties attached to C-1/C-14 of the [7.7] paracyclophane scaffold. Chemical structures of carbamidocyclophane A-U are described in Figure 1A.

#### Biological Activities

The methanol (MeOH) extract of *Nostoc* sp. CAVN 10 was active against different Gram-positive bacteria including drug-resistant strains and fungus as *Candida maltosa* SBUG 700, as well (Bui et al., 2007). Similarly, the MeOH extract of *Nostoc* sp. CAVN2 showed significant antimicrobial activity against methicillin-resistant *Staphylococcus aureus* (MRSA) and *Streptococcus pneumoniae* (Preisitsch et al., 2015). However, there was no effective activity against Gram-negative bacteria such as *Escherichia coli, Klebsiella pneumonia*, and *Pseudomonas aeruginosa*. Carbamidocyclophane A-C exhibited cytotoxicity against MCF-7 cells (breast adenocarcinoma cell line) and F1 cells (human amniotic epithelial cell line) with IC_50_ values 3.3, 4.2, and 5.1 μM, respectively (Bui et al., 2007). Carbamidocyclophane F and G both exhibited anti-proliferative activity against human cancer cell lines as MDA-MB-435 and HT-29 (Luo et al., 2014).

### Nostoflan

#### Origins

Nostoflan was isolated from *Nostoc flagelliforme*, a terrestrial cyanobacterium collected from Alxa, Inner Mongolia, China (Kanekiyo et al., 2005).

#### Isolation, Purification, and Detection Methods

Based on the bioassay guided isolation of compounds targeting molecules with activity against herpes simplex virus type 1 (HSV-1), the potent insoluble fraction was isolated. The potent fraction was subjected to ion-exchange column chromatography and gel filtration on Sepharose 6B. Finally, the strongest potent fraction (SI = 13,000) was subjected to HPLC system to obtain nostoflan (Kanekiyo et al., 2005).

#### General Chemical Structures

Nostoflan is an acidic polysaccharide with molecular weight of 2.11 × 10^5^. Nostoflan can be partially hydrolyzed into two oligosaccharides, PA-1 and PA-2. Specifically, the structures of PA-1 and PA-2 are β-D-GlcAp-(1 → 4)-Xyl-PA and β-D-GlcAp-(1 → 6)-β-D-Glcp-(1 → 4)-Gal-PA, respectively (Kanekiyo et al., 2005). Chemical structures of nostoflan and its derivatives are depicted in Figure 1B.

#### Biological Activities

Nostoflan exhibited promising antiviral activity against herpes simplex virus type 1 (HSV-1), HSV-2, human cytomegalovirus, and influenza A virus, whereas no activity was observed against adenovirus and coxsackie virus (Kanekiyo et al., 2005; Hayashi et al., 2008). The anti-herpetic effect of nostoflan was reported, whereas the effect was due to the binding property of compound with the virus, not by the penetration into host cell. So, nostoflan can be an effective anti-herpes molecule (Kanekiyo et al., 2007).

### Cylindrocyclophane and merocyclophane

#### Origins

Cylindrocyclophanes A, C, F and A_1_-A_4_, C_1_-C_4_, F_4_, and A_B4_ were isolated from the *Nostoc* sp. UIC 10022A (Chlipala et al., 2010). Merocyclophanes A and B were isolated from the *Nostoc* sp. UIC 10062, collected from Grand Mere State Park in Michigan (Kang et al., 2012). Merocyclophanes C and D were isolated from *Nostoc* sp. UIC 10110 (May et al., 2017).

#### Isolation, Purification, and Detection Methods

Solvent partition of extract, bioassay-guided fractionation, Diaion HP20, and RP HPLC were used to purify the target compounds. The initial organic extract from *Nostoc* sp. UIC 10022A was tested against the 20S proteasome and four cancer cell lines. Subsequent positive fractions were selected for Diaion VLC and semipreparative HPLC for further analysis. Finally, the chemical structures were predicted by ESI-TOF-MS and 1H NMR spectroscopy. The structures were confirmed as cylindrocyclophanes A, C, F along with 10 novel analogs: cylindrocyclophanes A1-A4, C1-C4, and F4 (Chlipala et al., 2010).

Solvent extraction of freeze-dried cells of *Nostoc* sp. UIC 10062 was tested for anti-proliferative activity against the HT29 human colon cancer cell line. Subsequently, potent fraction was eluted with an increasing gradient of isopropanol in H_2_O using Diaion HP20 resin. Liquid chromatography-mass spectroscopy (LC-MS) and UV absorbance were used for the analysis of active fractions eluted at 70 and 80% isopropanol. Finally, merocyclophanes A and B were purified by RP HPLC (Kang et al., 2012). Merocyclophane C and D were extracted with the solvent mixture and fractionated with Diaion HP20 VLC using gradient of isopropanol: H_2_O. These gradient eluents were used for bioassay against MDA-MB-435 cell lines, and the best fraction was used for further purification by RP-HPLC (May et al., 2017).

#### General Chemical Structures

Cylindrocyclophanes and merocyclophanes are the major classes of the [7,7] paracyclophanes varying in the presence/absence of an α or β methyl substituent. Cylindrocyclophanes contain β-branched methyls at C-2/15, whereas merocyclophanes A and B contain α-branched methyls at C-1/14. Merocyclophanes C and D are analogs of the α-methyl branched merocyclophane providing evidence that [7,7] paracyclophanes are structurally diverse compounds (May et al., 2017). Chemical structures of cyclindrocyclophane, merocyclophane, and their derivatives are illustrated in Figures 1C,D.

#### Biological Activities

Cylindrocyclophanes A_1_-A_4_, C_4_, C_2_, F_4_, AB_4_, A, C, and F were tested for inhibitionary effects against the 20S proteasome. These compounds displayed different ranges of activities in which variants A_2_-A_4_ were the most potent compounds with IC_50_ = 2.55–3.93 μM (Chlipala et al., 2010).

Merocyclophanes A and B were evaluated for antiproliferative activity against the HT-29 human colon cancer cell line, whereas the results exhibited significant activity with IC_50_ values 3.3 and 1.7 μM, respectively. Both merocyclophanes A and B displayed similar levels of antiproliferative activity equivalent to cylindrocyclophanes A-F against various cell lines (Kang et al., 2012). Merocyclophanes C and D inhibited proliferation of MDA-MB-435 cell line with IC_50_ values 1.6 and 0.9 μM, respectively (May et al., 2017).

### Scytonemin and Reduced Scytonemin

#### Origins

Scytonemin and reduced scytonemin, are hydrophobic pigment isolated from the terrestrial cyanobacterium *Nostoc commune* (Matsui et al., 2012; Itoh et al., 2013).

#### Isolation, Purification, and Detection Methods

The dried algal powder was extracted with ethanol, and fractionated with silica gel VLC using a mixture of n-hexane/ethyl acetate as eluent. The products were collected in 8 fractions. The bioactive fraction was repeatedly subjected to different column chromatographic (CC) techniques using silica gel CC, Sephadex LH-20 CC, and octadecylsilyl (ODS) CC to yield scytonemin. Further purification of bioactive sub-fraction using ODS CC and elutin by chloroform/methanol yielded reduced scytonemin (Itoh et al., 2013).

#### General Chemical Structures

Structurally, scynonemin contains indolic and phenolic subunits constituting a ring structure termed as the scytoneman skeleton. Structures of scytonemin and reduced scytonemin are described in Figure 1E.

#### Biological Activities

Scytonemin provided significant protection to cyanobacteria against ultraviolet radiation. Scytonemin were reported as slow-acting radical scavenger based on 2,2′-azino-bis(3-ethylbenzothiazoline-6-sulphonic acid) (ABTS) bioassay (Matsui et al., 2012). Reduced scytonemin significantly inhibited growth of human leukemia Jurkat cell in a concentration-dependent manner. The induction of type II programmed cell death (autophagic cell death) and ROS production is major MOA of these molecules (Itoh et al., 2013).

Both reduced scytonemin and scytonemin suppressed NO production induced by LPS/IFN γ. The reduced scytonemin also exhibited the anti-inflammatory effects mediated by ROS/PI3K/Akt and p38 MAPK/Nrf2 signaling pathways (Itoh et al., 2014a).

### Cryptophycin

#### Origins

Cryptophycin−38, −326, −327, −46, −175, and −176 were isolated from *Nostoc* sp. GSV 224, a terrestrial cyanobacterium (Subbaraju et al., 1997; Chaganty et al., 2004). Recently, numerous synthetic cryptophycin analogs have been generated and subjected to structure-activity-relationship (SAR) studies targeting to improve tumor selectivity and water solubility (Cazzamalli et al., 2018; Figueras et al., 2018).

#### Isolation, Purification, and Detection Methods

Lyophilized *Nostoc* sp. GSV 224 was extracted with 5:1 MeCN-CH_2_Cl_2_. The extract was subjected to reversed-phase flash column chromatography and HPLC for purification of the compound (Subbaraju et al., 1997).

#### General Chemical Structures

Cryptophycins belong to 16-membered macrocyclic depsipeptides. The core structure of cryptophycin consists of 4 units. The 4 units can be categorized as two hydroxy acids *viz*. 7,8-epoxy-5-hydroxy-6-methyl-8-phenyl-2-octenoic acid (unit A), 2-hydroxy-4-methylvaleric acid (unit D) and two amino acids, *viz*. 3-(3-chloro-4-methoxy-pheny1)alanine (unit B) and 3-amino-2-methylpropionic acid (unit C). The substitution of various residues in each unit can generate diverse cryptophycin derivatives. For instance, cryptophycin-175 has characteristics presence of two chlorines and a methoxyl group on the aromatic ring of unit B (Subbaraju et al., 1997). In other hand, cryptophycin-38 is a stereoisomer of cryptophycin-1 containing a *S, S* epoxide group in unit A. Similarly, cryptophycin-326 is an analog of cryptophycin-21 but it has two chlorines ortho to the methoxy group in unit B (Chaganty et al., 2004). Chemical structures of cryptophycin and its analogs are shown in Figure 1F.

#### Biological Activities

Cryptophycins are potent cytotoxic macrocyclic depsipeptides. They interact with tubulin and prevent microtubules from forming correct mitotic spindles. These cascades of events cause cell-cycle arrest and apoptosis. Cryptophycin-52 was investigated in phase II clinical trials but failed because of its high neurotoxicity (Weiss et al., 2017).

Cryptophycin demonstrated cytotoxicity against various human tumor cell lines as KB, LoVo, and SKOV3. The IC_50_ values cryptophycin-176 against these cell line was in the range of 1.3–1.6 × 10^−2^ μM (Subbaraju et al., 1997). The IC_50_ values cryptophycin-38 against human tumor cell line KB was 54.7 × 10^−2^ μM (Chaganty et al., 2004). Different analogs of cryptophycin progressed to preclinical and clinical trials (Edelman et al., 2003; Liang et al., 2005), hence cryptophycin are the most important natural products with pharmaceutical applications with cynobacterial origin.

### Nostocyclopeptide

#### Origins

Nostocyclopeptides A1 and A2 were isolated from the *Nostoc* sp. ATCC53789 (Golakoti et al., 2001). Nostocyclopeptides M1 was isolated from the culture of the *Nostoc* sp. XSPORK 13A (Jokela et al., 2010).

#### Isolation, Purification, and Detection Methods

Solvent extract of *Nostoc* sp. ATCC53789 was fractionated by reversed-phase flash chromatography. The fraction containing nostocyclopeptides A1 and A2 was subjected into RP-HPLC to obtain purified compounds (Golakoti et al., 2001).

For isolation of nostocyclopeptides M1, freeze-dried biomass was extracted with a solvent, filtered and passed through two SPE cartridges. Then MeOH was used as eluent to obtain the semi-pure fraction, which was later subjected into the HPLC system for purification of Ncp-M1 (Jokela et al., 2010).

#### General Chemical Structures

Nostocyclopeptide A1 contains seven amino acid units (Tyr, Gly, Gln, Ile, Ser, 4-MePro, and Leu). Nostocyclopeptide A2, differs from nostocyclopeptide A1 due to the presence of a modified phenylalanine unit with an imine linkage connected to the *C*-1 of the modified phenylalanine and the nitrogen of the tyrosine unit (Golakoti et al., 2001).

Structurally, nostocyclopeptide M1 contains seven amino acids (Tyr1-Tyr2-d-HSe3-l-Pro4-l-Val 5-(*2S,4S*)-4-MPr6-Tyr7) with characteristics imino linkage between Tyr1 and Tyr7 (Herfindal et al., 2011). Structures of nostocyclopeptide and its derivatives are illustrated in Figure 1G.

#### Biological Activities

The nostocyclopeptide A1 and A2 showed weak cytotoxicity against cell lines as KB (a human nasopharyngeal carcinoma) and LoVo (a human colorectal adenocarcinoma) with an IC_50_ value 1 μM. These compounds had no effect against the pathogenic fungi and bacteria tested in the study (Golakoti et al., 2001). Nostocyclopeptide M1 was reported as non-toxic and anti-toxin of microcystin, as it prevented microcystin/nodularin-induced apoptosis in rat hepatocytes (Jokela et al., 2010). Furthermore, the compound acted as a potent and non-toxic inhibitor of organic anion transporters OATP1B1 and OATP1B3 expressed in HEK293 cells (Herfindal et al., 2011).

### Hassallidin

#### Origins

Anti-fungal glycolipopeptide hassallidin was isolated from various cyanobacteria such as *Anabaena* spp. strain (BIR JV1 and HAN7/1), *Nostoc* spp. strain (6sf Calc and CENA 219) and *Hassallia* sp. These strains were isolated from brackish and freshwater samples collected in Brazil, the Czech Republic, and Finland, respectively (Shishido et al., 2015).

#### Isolation, Purification, and Detection Methods

One hundred and ninety four members of genera *Cyanobacteria* isolated from diverse habitats were screened for bioactivity guided isolation of antifungal compounds against *Candida albicans* and/or *Aspergillus flavus* using a disc diffusion assay. The freeze-dried cells of cyanobacteria were extracted with methanol and tested for antifungal activities, whereas two *Nostoc* strain (CENA 219 and calcicula 6 sf Calc) exhibited activity against both fungus. The antifungal compounds were analyzed using LC-MS. Fourteen different congeners of hassallidin were detected in the methanol extract of *Nostoc* sp. CENA 219. The prime hassallidin variants found in the extract of *Nostoc calcicula* 6 sf Calc were hassallidin 12, hassallidin 14, and hassallidin 15. These variants differed in the nature of sugars (pentose, deoxyhexose, hexose, acetylated hexose, and N-acetylhexosamine), the composition of the aglyconic peptide core structure and the fatty acid moiety. The relative amounts of hassallidins were roughly estimated from the TIC chromatograms whereas variants 11, 15, and 26 were the most abundant hassallidins in *Nostoc* sp. CENA 219.

#### General Chemical Structures

Hassallidin, a cyclic glycosylated lipopeptide, contains structural variations such as presence of dihydroxy fatty acids and complex glycosylated patterns by monosaccharide moieties (pentose, deoxyhexose, hexose, acetylated hexose, and *N*-acetylhexosamine) that are attached to the aglyconic peptide core structure. Initially, the structure of hassallidins A and B were established to containdihydroxy fatty acid side chain with mannose and rhamnose, respectively. However, new types of hassallidin were reported to contain five optional monosaccharides: *N*-acetylhexosamine (M1), pentose (M1 and M2), hexose (M1 and M3), deoxyhexose (M1 or M2), or acetylhexose with different degrees of acetylation (M3). *N*-acetylhexosamine was only isolated from the Brazilian *Nostoc* sp. CENA 219 (Vestola et al., 2014; Shishido et al., 2015) (Figure 1H). Chemical structures of hassallidin and its derivatives are given in Figure 1H.

#### Biological Activities

Neuhof et al. (2005) reported hassallidin variants as a potent anti-fungal agent. The specific anti-fungal activity was in the range of MIC 3.42 μM against *Aspergillus fumigatus* and *Candida albicans* (Neuhof et al., 2005). However the antifungal activity of hassallidin B against the strains of *Candida* species and *Cryptococcus neoformans* was nearly identical to that of hassallidin A (Neuhof et al., 2006). However, hassallidin D showed higher anti-fungal activity (MIC ≤ 1.5 μM) than hassallidin A and B (Vestola et al., 2014). In addition, hassallidins A and B exhibited cytotoxic activity against human Jurkat ATCC-TIB-152 (a type of acute T-cell leukemia) and L929 (a type of murine aneuploid fibrosarcoma cell line) (Shishido et al., 2015).

Additional information on diverse secondary metabolites characterized from Nostoc sp. and their bioactivities are described in Table 1 and Figure 1.

**Table 1:** Lists of compounds from *Nostoc* and biological activities.

**No**	**Chemical names**	**Cyanobacterial strains**	**Description of bioactivities**	**References**
1	Comnostins A-E	*N. commune*	Anti-bacterial (*E. coli, S. epidermidis, B. cereus*). Comnostin B showed cytotoxicity to KB, Caro-2 cells as well as molluscicidal activity against *Biomphalaria glabrata*	Jaki et al., 2000b
2	Pseudospumigins A-F	*Nostoc* sp. CENA543	Protease inhibitors (trypsin): IC_50_ 4.5 μM for pseudospumigin A	Jokela et al., 2017
3	Nostocionone	*N. commune* Vauch.	Inhibited *P. acnes* growth (suppressed hk *P. acnes*-induced nitric oxide (NO) production through the suppression of inducible NO synthase expression, following inactivation of NF-κB): IC_50_ 60 μM	Itoh et al., 2014b
4	(9*Z*,12*Z*)-9,12,15-hexadecatrienoic acid (n-1 fatty acid)	*N. verrucosum*	Antibacterial activity: inhibit the growth of *S. aureus* at MIC 256 μM	Oku et al., 2014
5	Polysaccharide from *N. commune* Vauch. (NVPS)	*N. commune* Vauch.	Cytotoxicity *in-vitro* (MCF-7 and DLD1 cells): the IC_50_ values NVPS against MCF-7 and DLD1 cell lines were 0.28 and 0.04 μM, respectively	Guo et al., 2015
6	Nostocyclyne A	*Nostoc* sp. TAU strain IL-220	Anti-microbial activity (MIC values against *S. aureus* and *B. subtilis* of 12.5 and 10 μg/disk, respectively) and weak inhibition of photosynthesis in green alga (5% at 292.4 μM)	Ploutno and Carmeli, 2000
7	Swinholides	*Nostoc* sp. UHCC 0450.	Anti-fungal activity *(A. flavus)*	Humisto et al., 2017
8	Prenostodione	*Nostoc* sp. (TAU strain IL-235)	UV-absorbing activity	Ploutno and Carmeli, 2001
9	EMTAHDCA (9-ethyliminomethyl-12-(morpholin-4-ylmethoxy)-5, 8, 13, 16–tetraaza–hexacene-2, 3 dicarboxylic acid)	*Nostoc* sp. MGL001	Growth inhibiting effects mainly against the gram negative bacterial strains, ability to bind 30S ribosomal fragment (PDB ID: 1YRJ, 1MWL, 1J7T, and 1LC4) and OmpF porin protein (4GCP, 4GCQ, and 4GCS)	Niveshika et al., 2016
10	A diterpenoid (8-[(5-carboxy-2,9-epoxy)benzyl]-2,5-dihydroxy-1,1,4a,7,8 pentamethyl-1,2,3,4,4a,6,7,8,9,10,10a dodecahydrophenanthrene), an anthraquinone (1,8-dihydroxy-4-methylanthraquinone) and an indane derivative (4-hydroxy-7-methylindan-1-one).	*N. commune* (EAWAG 122b)	Anti-bacterial activity against *S. epidermidis* and *B. cereus*	Jaki et al., 2000a

### Lyngbya

*Lyngbya* species form long, unbranching filaments incorporated into a rigid mucilaginous sheath. They form the basic component of the oceanic food chain by intermixing with other phytoplankton species. The toxin of *Lyngbya* can cause the human skin irritation called as seaweed dermatitis (Lee, 2018).

### Lagunamide

#### Origins

Lagunamides A-C were isolated from the *Lyngbya majuscula* from Pulau Hantu Besar, Singapore (Tripathi et al., 2010, 2011). But lagunamide D was mined from a collection of marine cyanobacteria (a mixture mainly consisting *of Dichothrix* sp. and *Lyngbya* sp. with minor presence of *Rivularia* sp.) in Florida (Luo et al., 2019).

#### Isolation, Purification, and Detection Methods

Various isolation methods were used to isolate different variants of lagunamide by extraction with CHCl_3_/MeOH and fractionation by VFC in combination with bioassay-guided screening. The bioactive fractions were subjected to SEP PAK C18 solid-phase extraction, followed by RP-HPLC to yield lagunamides A-C (Tripathi et al., 2010, 2011). Similarly, a mixture of EtOAc–MeOH (1:1) was used for extraction of non-polar fraction, which was subsequently partitioned between EtOAc and H_2_O to yield two crude fractions. It was further fractioned by C18 SPE followed by RP-HPLC to yield two semi-pure fractions. It was found that there was interconversion between these two molecules after second round of HPLC purification. By optimation of several factors, such as HPLC solvents, the time of the exposure of compound to air, temperature, etc., two lagunamide derivatives D and D' were successfully isolated (Luo et al., 2019).

#### General Chemical Structures

Lagunamides are class of cyclodepsipeptides with hybrid core structure consisting of polypeptide(substructures A) and polyketide (substructures B). For example, lagunamides A has substructure A with six amino acid residues: *N*-methylalanine (*N*-Me-Ala)- isoleucine (Ile)-*N*-methylglycine (*N*-Me-Gly)-*N* methylphenylalanine (*N*-Me-Phe)-alanine (Ala) and 2-hydroxyisoleucic acid (Hila), whereas substructure B (C33-C42) contains a hydroxy group at C-37 and a methyl group at C-43 (Tripathi et al., 2010).

**Figure 2:**
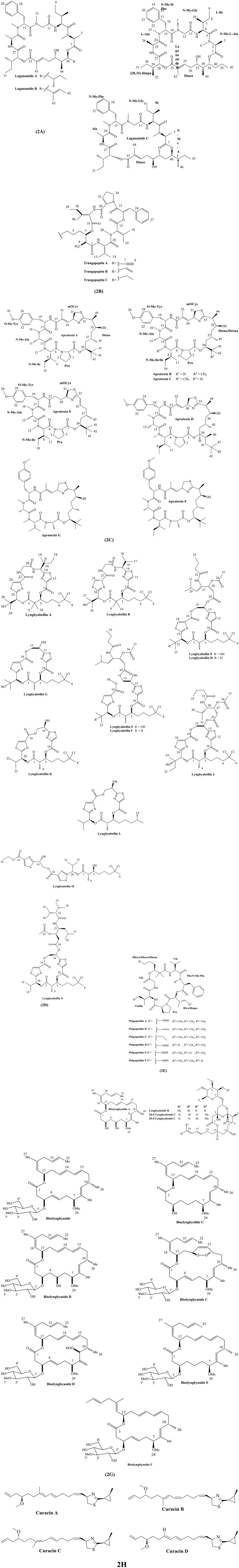
Chemical structures of bioactive molecules from *Lyngbya*. **(A)** Lagunamides, **(B)** Trungapeptins, **(C)** Apratoxins, **(D)** Lyngbyabellins, **(E)** Pitipeptolides, **(F)** Lyngbyalosides, **(G)** Biselyngbyasides, **(H)** Curacins.

Substructure A of lagunamide C includes five proteinogenic amino acids, *viz N*-methyl-alanine (C-1–C-4), isoleucine (C-5–C-10), *N*-methyl-glycine (C-11–C-13), *N*-methyl-phenylalanine (C-14–C-23) and alanine (C-24–C-26), while substructure B is polyketide segment containing 5,8-dihydroxy-2,6,9-trimethyl-undec-2-enoic acid (Dtuea) moieties (Tripathi et al., 2011). Substructure A of lagunamide D is similar to lagunamide C but polyketide portion of lagunamide D is α-hydroxy acid 2-hydroxy-3-methylpentanoic acid (Hmpa) (Luo et al., 2019). Chemical structures of diverse lagunamides are depicted in Figure 2A.

#### Biological Activities

Lagunamides A and B showed antimicrobial activity against *Pseudomonas aeruginosa* strain PA01 and malarial parasite *Plasmodium falciparum* NF54 with IC_50_ values 0.19 and 0.91 μM, respectively (Tripathi et al., 2010). Meanwhile, lagunamide C was reported with potent cytotoxicity against various types of cancer cell lines, such as P388, A549, PC3, HCT8, and SK-OV3 with IC_50_ values ranging from 2.1–24.4 × 10^−2^ μM (Tripathi et al., 2011). Lagunamide A and D exhibited antiproliferative activity even in low-nanomolar range against A549 human lung adenocarcinoma cells with an IC_50_ value of (6.7 ± 2.2) × 10^−3^ and (7.1 ± 1.7) × 10^−3^ μM, respectively (Luo et al., 2019). It was also reported that lagunamides A had selective cytotoxicity against a series of cancer cell lines, whereas HCT8 was the most sensitive cell line. The preliminary data from a series of biochemical experiments suggested that the cytotoxic effects of the lagunamides in HCT8 and MCF-7 cell lines is mediated by the intrinsic apoptotic pathway (Tripathi et al., 2012). Recently, Huang et al. (2016) deduced that the *R* configuration of lagunamide A at C-39 position was the structural scaffold responsible for anti-cancer activity through the assessment of cytotoxicity against various cancer cell lines as A549, HeLa, U2OS, HepG2, BEL-7404, BGC-823, HCT116, MCF-7, HL-60, and A375. More apparently, investigation of the molecular mechanism of lagunamide A revealed that it induced caspase-mediated mitochondrial apoptosis (Huang et al., 2016).

### Trungapeptin

#### Origins

Trungapeptins A-C were isolated from the marine cyanobacterium *Lyngbya majuscula* collected from Trung Province, Thailand (Bunyajetpong et al., 2006).

#### Isolation, Purification, and Detection Methods

The organic extract of the culture was initially separated by silica gel liquid chromatography. Bioassay-guided fractionation by Sephadex LH-20, RP-Sep-Pak, and RP-HPLC yielded colorless oils of trungapeptins A-C (Bunyajetpong et al., 2006).

#### General Chemical Structures

Trungapeptins A-C are class of depsipeptide containing the unique 3-hydroxy 2-methyl-7-octynoic acid (Hmoya), 3-hydroxy2-methyl-7 octenoic acid (Hmoea), and 3-hydroxy-2-methyl-7-octanoic acid (Hmoaa) units, respectively. Trungapeptin A contains five amino acids: valine (Val), *N*-methylvaline (*N*-Me-Val), proline (Pro), and isoleucine (Ileu); a 3-phenyllactic acid (Pla) and a 3-hydroxy-2-methyl-7-octynoic acid (Hmoya) with the sequence as Hmoa/Val/N-MeVal/Pla/Pro/Ileu. Trungapeptins B and C vary due to the presence of the octenoic and octanoic acid residues in their structures, respectively (Bunyajetpong et al., 2006). In addition, the absolute configuration of the Hmoa unit in trungapeptin C is assigned as (*2S,3R*)-Hmoa (Gupta et al., 2016). Structure of trungapeptin A-C are illustrated in Figure 2B.

#### Biological Activities

Trungapeptin A showed mild brine shrimp toxicity and mild ichthyotoxicity but no cytotoxicity was observed against KB and LoVo cells. Due to a limited supply of compounds trungapeptin B-C, their biological activities could not be evaluated (Bunyajetpong et al., 2006).

### Apratoxin

#### Origins

Apratoxin A was isolated from the marine cyanobacterium *Lyngbya majuscule*, while apratoxin B and C were obtained from marine cyanobacterium *Lyngbya* sp. (Luesch et al., 2001b, 2002c). Apratoxin D was isolated from *L. majuscula* and *L. sordida*, while apratoxin E, F, and G were isolated from the marine *Lyngbya bouillonii* in different geographical locations (Gutierrez et al., 2008; Matthew et al., 2008; Tidgewell et al., 2010).

#### Isolation, Purification, and Detection Methods

Different types of solvents were used to screen the targeted compounds in total extract obtained from the freeze-dried biomass of *Lyngbya*. Further, it was fractionated and tested for cytotoxicity. The bioactive fraction was subjected to RP-HPLC to yield apratoxin A and B (Luesch et al., 2001b, 2002c). For isolation of apratoxin C, the lipophilic partition was extracted from freeze-dried biomass followed by fractionation between *n*-BuOH and H_2_O. The concentrated *n*-BuOH phase was subsequently subjected to silica gel chromatography and semipreparative reversed-phase HPLC to yield apratoxin C (Luesch et al., 2002c).

Bioassay-guided partition of extract from *L. majuscula* and *L. sordida* was performed to screen the bioactive fractions. The fractions were purified by RP-HPLC to obtain apratoxin D from *L. majuscula* and *L. sordida*, respectively (Gutierrez et al., 2008). The crude extract of *L. bouillonii* was fractionated by normal-phase chromatography and tested for cytotoxicity. Finally, apratoxin E present in the most cytotoxic fraction was purified by RP-HPLC (Matthew et al., 2008). Similarly, the crude extract of *L. bouillonii* was fractionated by VLC chromatography and sequential isocratic RP-HPLC yielded apratoxin F and G (Tidgewell et al., 2010).

#### General Chemical Structures

Apratoxin derivatives are class of cyclodepsipeptide containing hybrid peptide-polyketide structure. They contain a thiazoline ring flanked by polyketide portions, whereas one of which possess an unusual methylation residue. Apratoxin A structurally consists of an α, β-unsaturated modified cysteine residue (moCys, C-27–C-32), one regularamino acid unit (proline) and three methylated amino acid moieties (*O*-methyltyrosine, *N*-methylalanine, *N*-methylisoleucine). Thelinear sequence is (*N*-Me-Ile)-(*N*-Me-Ala)-(*O*-Me-Tyr)-moCys-Dtena-Pro (Luesch et al., 2001b).

Apratoxin B contains one methylene unit less than apratoxin A and an isoleucine residue instead of the N-methyl isoleucine moiety. Generally, variant A, B, and C differ in the substitution pattern of methyl residue in 3,7-dihydroxy-2,5,8,8-tetramethylnonanoic acid portion C-33 –C-45 (Dtena)/3,7-dihydroxy-2,5,8-trimethylnonanoic acid (Dtrina) and Ile/*N*-Me-Ile units (Luesch et al., 2001b, 2002c). Particularly, apratoxin D contains a new polyketide moiety, 3,7-dihydroxy-2,5,8,10,10-pentamethyl undecanoic acid, while retaining the same sequence of amino acid residues as apratoxins A and C (Gutierrez et al., 2008).

Apratoxin E contains peptide components (*O*-Me-Tyr, *N*-Me-Ile, *N*-Me-Ala, and Pro) and polyketide fragments (C-27–C-31 and C-33–C-45) similar to apratoxin A, however it has a lower degree of C-methylation. Apratoxin E consists of a 7-hydroxy-5,8,8-trimethyl-2-nonenoic acid residue (C-33–C-45). The close similarity of apratoxin E and E-dehydroapratoxin A indicates the existence of same relative configuration in the polyketide chain (Matthew et al., 2008).

Apratoxin F differs from apratoxin A by a single carbon atom and therefore possess one less degree of unsaturation. Moreover, apratoxin G differs from apratoxin F by a single methylene unit. Apratoxin F contains L-*N*-Me-Ala, L-*O*-Me-Tyr, and L-*N*-Me-Ile, while apratoxin G contains L-*N*-Me-Ala, L-*O*-Me-Tyr, and L-*N*-Me-Val (Tidgewell et al., 2010). Structures of apratoxin A-G are shown in Figure 2C.

#### Biological Activities

Apratoxin A was reported to have cytotoxicity against human tumor cell lines (KB and LoVo cancer cells) with IC_50_ value ranging from 3.6–5.2 × 10^−4^ μM (Luesch et al., 2001b).

It was reported that Apratoxin B and C had cytotoxicity against KB and LoVo cell lines with IC_50_ in the range of 1.08–2.13 × 10^−2^ μM, and 0.73–1.0 × 10^−3^ μM, respectively, which is relatively lower cytotoxicity than apratoxin A (Luesch et al., 2002c). In *in vitro* assays, apratoxin D showed potent cytotoxicity against human lung cancer cell (H-460) with an IC_50_ value 2.6 × 10^−3^ μM (Gutierrez et al., 2008). Similarly, apratoxin E demonstrated cytotoxicity against HT29, HeLa, and U2OS cell lines with an IC_50_ value in ranging of 2.1–7.2 × 10^−2^ μM (Matthew et al., 2008). Apratoxin F and G also showed potent cytotoxicity against H-460 cancer cell with IC_50_ values 2 × 10^−3^ and 14 × 10^−3^ μM, respectively (Tidgewell et al., 2010).

### Lyngbyabellin

#### Origins

Lyngbyabellins A, B, and E-I are secondary metabolites isolated from the *Lyngbya majuscula* (Luesch et al., 2000a, 2002b; Bingnan et al., 2005). Moreover, lyngbyabellins K-N and 7-*epi*-lyngbyabellin L are derived from the marine cyanobacterium *Moorea bouillonii* (Choi et al., 2012).

#### Isolation, Purification, and Detection Methods

Isolation of lyngbyabellin A was carried out by selecting the less cytotoxic fraction. The selected fraction was subsequently subjected to semipreparative RP-HPLC using an isocratic system of 80% aqueous MeCN (Luesch et al., 2000b). Similarly, lyngbyabellin B was isolated from the less polar fraction than lyngbyabellin A (Luesch et al., 2000a).

The extract of *L. majuscula* biomass was fractionated by silica gel vacuum liquid chromatography and subsequently potent toxic fractions against brine shrimp model were selected for SPE cartridge. Finally, RP-HPLC was used to recollect the purified compounds, which were later confirmed as five new lyngbyabellin analogs E-I (Bingnan et al., 2005). In another study by Choi et al. (2012), the fractions having moderate anti-cancer activity against human lung cancer cells (H-460) was purified by RP-HPLC and reported as lyngbyabellin K, M, and two epimers of lyngbyabellin L. Further, the mixed epimers were separated by chiral HPLC and confirmed as pure lyngbyabellin L and 7-*epi*-lyngbyabellin L (Choi et al., 2012).

#### General Chemical Structures

Lyngbyabellins are a group of lipopeptide characterized by the presence of two thiazole rings, and a chlorinated and 2-methyl octanoate residue. For instance, lyngbyabellin A consists of two chlorine atoms and two 2-alkylthiazole-4-carboxylic acid units (C-11 to C-14 and C-22 to C-25) (Luesch et al., 2000b). Both lyngbyabellin B and A possess 7,7-dichloro-3-acyloxy-2,2-dimethyloctanoic acid and α, β-dihydroxy-isovaleric acid residues, but lyngbyabellin B has one less carbon atom than lyngbyabellin A (Luesch et al., 2000a). Lyngbyabellin H is analog of lyngbyabellin E without the hydroxyl group in C-23 position. The structural pattern is also similar in lyngbyabellin I and F (Bingnan et al., 2005).

Lyngbyabellin K contains two chlorine atoms in the molecule, two 2,4-disubstituted thiazole rings, and amide functionalities. Lyngbyabellin L consists of two extended 2,4-disubstituted thiazole units, a HIVA unit, and a 1,2-dihydroxyethyl moiety. Both lyngbyabellin L and 7-*epi*-lyngbyabellin L contain a single chlorine atom, but later is stereoisomer of the former at the C-7 position. Lyngbyabellin M belongs to the same enantiomeric series as lyngbyabellin K (Choi et al., 2012). Structures of lyngbyabellin A-M are shown in Figure 2D.

#### Biological Activities

Lyngbyabellin A was toxic to mice under *in vivo* test. Lyngbyabellin A had moderate cytotoxicity against KB cells and LoVo cells (Luesch et al., 2000b). Lyngbyabellin B exhibited cytotoxicity against KB and LoVo cells with IC_50_ values 0.15 and 1.22 μM, respectively. Lyngbyabellin B was slightly more cytotoxic in *in vitro* trials than lyngbyabellin A (Luesch et al., 2000a).

All five lyngbyabellins E-I showed promising cytotoxicity to NCI-H460 human lung tumor and neuro-2a neuroblastoma cell lines in mouse (Bingnan et al., 2005). Lyngbyabellin N showed strong yet variable cytotoxicity, while lyngbyabellins K, L, M, and 7-*epi*-lyngbyabellin L were inactive (Choi et al., 2012).

### Pitipeptolides

#### Origins

Pitipeptolides A-B and C-F were isolated from the marine cyanobacterium *Lyngbya majuscula* at Guam (Luesch et al., 2001a; Montaser et al., 2011b).

#### Isolation, Purification, and Detection Methods

Crude extract from *L. majuscula* was solvent partitioned and subjected to silica chromatography resulting in a mixture of pitipeptolides A and B. Later it was subjected to normal-phase HPLC, yielding both pitipeptolides A and B (Luesch et al., 2001a). After several steps of extraction with EtOAc-MeOH mixtures and *n*-BuOH, the extract was fractionated by silica gel chromatography and subsequently subjected to RP HPLC to yield pitipeptolides C-F (Montaser et al., 2011b).

#### General Chemical Structures

Pitipeptolides are cyclodepsipeptides structurally characterized by the presence of 2,2-dimethyl-3-hydroxy-7-octynoic acid residue in pitipeptolide A and a 2,2-dimethyl-3-hydroxy-7-octenoic acid residue in pitipeptolide B. Particularly, pitipeptolide A consists of seven units including *N* methylphenylalanine (*N*-MePhe), glycine (Gly), proline (Pro), valine (Val), isoleucine (Ile), 2-hydroxy-3-methylpentanoic acid (Hmp), and a 2,2-dimethyl-3-hydroxy-7-octynoic acid (Dhoya). In pitipeptolides B, Dhoya unit is substituted by a 2,2-dimethyl-3-hydroxy-7-octenoic acid (Dhoea) unit (Luesch et al., 2001a).

Pitipeptolide C is a tetrahydro- analog of pitipeptolide A containing the completely saturated fatty acid derived unit 2,2-dimethyl-3-hydroxy octanoic acid (Dhoaa). Pitipeptolide D is a analog of pitipeptolide A with a lower degree of methylation and the Phe residue in pitipeptolide D lacks the *N*-Me modification. Pitipeptolides E and F have the same molecular formula as pitipeptolide D and resemble as analog of pitipeptolide A containing one less methylene group. Pitipeptolides E has Hmpa → Hiva displacement, while pitipeptolide F has Ile → Val displacement in comparison to pitipeptolide A (Montaser et al., 2011b). Chemical structures of pitipeptolide A-C are depicted in Figure 2E.

#### Biological Activities

Pitipeptolides A, B, C, and E were reported as a potent growth inhibitor of two *Mycobacterium tuberculosis* strains (ATCC 25177 and ATCC 35818). Particularly, pitipeptolide F exhibited the highest anti-mycobacterial activities in the disc diffusion assay (Luesch et al., 2001a; Montaser et al., 2011b). Pitipeptolides A, B, and C-F also exhibited cytotoxicity against HT 29 colon adenocarcinoma and MCF-7 breast cancer cell lines (Montaser et al., 2011b). In addition, pitipeptolides A and B exhibited weak cytotoxicities against LoVo cells (Luesch et al., 2001a).

### Lyngbyaloside

#### Origins

Lyngbyaloside B was isolated from the cyanobacterium *Lyngbya* sp. NIH309 (Luesch et al., 2002b) while 2-*epi*-lyngbyaloside, 18E- and 18Z lyngbyalosides C were isolated from the marine cyanobacterium *Lyngbya bouillonii* (Matthew et al., 2010).

#### Isolation, Purification, and Detection Methods

Lyngbyaloside B was purified from the lipophilic extract of *Lyngbya* sp. biomass using silica gel chromatography and semipreparative reversed-phase HPLC (Luesch et al., 2002b). Solvent partition of extract from *L. bouillonii* was performed to collect 16 fractions. The fraction 5–7 were subjected to the semipreparative HPLC system to obtain a mixture of lyngbyalosides. Finally, the mixture was purified by reversed-phase semipreparative HPLC to obtain different analogs of lyngbyalosides (Matthew et al., 2010).

#### General Chemical Structures

Lyngbyaloside B isglycoside-polyketide structure. Lyngbyaloside B consists of seven methylenes and six methyls in which two of the later exist in the oxygenated form. Eight of the 14 methines were oxygenated and four other methines were olefinic. Hexopyranoside structure and the two oxygenated methyl groups were located at C-3′ and C-4′ in lyngbyaloside B, respectively (Luesch et al., 2002b).

2-*epi*-lyngbyaloside has the same planar structure as tricyclic glycoside macrolide lyngbyaloside. 18E–lyngbyaloside C is closely related to lyngbyaloside B with same molecular formula. It contains an ester moiety, a hemiketal, a hexapyranoside group, and a terminally brominated conjugated hexadiene side chain in its structure (Matthew et al., 2010). Chemical structures of lyngbyaloside derivatives are depicted in Figure 2F.

#### Biological Activities

Lyngbyaloside B showed slight *in vitro* cytotoxicity against KB cells and even lesser effect on LoVo cells with an IC_50_ value 4.3 and 15 μM, respectively (Luesch et al., 2002b).

Lyngbyaloside and its 2-epimer lyngbyaloside showed even weaker cytotoxicity against HT29 colorectal adenocarcinoma and HeLa cervical carcinoma cells with an IC_50_ value in the range of 33–38 μM (Matthew et al., 2010).

### Biselyngbyaside

#### Origins

Biselyngbyolide derivatives A-F were isolated from the marine cyanobacterium *Lyngbya* sp., in different locations of Japan (Teruya et al., 2009; Morita et al., 2012a,b; Watanabe et al., 2015).

#### Isolation, Purification, and Detection Methods

Bioassay-guided fractionation of the cytotoxic constituents of *Lyngbya* sp. against HeLa cells in combination with reversed-phase HPLC was used to yield diverse variants of biselyngbyaside (Teruya et al., 2009; Morita et al., 2012a,b; Watanabe et al., 2015).

#### General Chemical Structures

Biselyngbyasides is 18-membered macrolide containing lactone ring as tedanolide and 13-deoxytedanolide between C-1 and C-17 to form the core structure. Biselyngbyaside derivatives are diversified by the addition of 3-*O*-methyl glucoside moiety at C-2 and methyl groups (Teruya et al., 2009). For instance, substituted groups in biselyngbyolide A consist of an aliphatic methyl, three vinyl methyls, an *O*-methyl group, an aliphatic methine, four oxymethines, and eight olefinic methines (Morita et al., 2012a). The presence of sugar in the form of β-anomeric structure differentiate between biselyngbyasides B and A. In addition, biselyngbyaside C is similar to the biselyngbyaside except for the presence of an endoperoxide linkage at C-12 and C-15 with *cis*-configuration (Morita et al., 2012b). Biselyngbyaside F was determined as a *trans*-isomer of C18 olefin of biselyngbyaside (Watanabe et al., 2015). Chemical structure of biselyngbyolide derivatives A–F are shown in Figure 2G.

#### Biological Activities

Biselyngbyaside A showed broad-spectrum cytotoxicity against human cancer cells, for example, HeLa S3 cells with an IC_50_ value 0.17 μM (Teruya et al., 2009). Biselyngbyolide B, C, E, and F exhibited the growth inhibition of HeLa and HL-60 cells. Moreover, biselyngbyolide C was found to induce ER stress and apoptosis in HeLa cells (Watanabe et al., 2015). Biselyngbyolide A showed cytotoxicity against HeLa S3 and HL-60 human leukemia cells with IC_50_ values 0.14 and 0.012 μM, respectively (Morita et al., 2012b). Biselyngbyaside highly inhibited receptor activator of nuclear factor(NF)-kB ligand (RANKL)-induced osteoclastogenesis in mouse monocytic RAW264 cells and primary bone marrow-derived macrophages at a low concentration (Yonezawa et al., 2012).

### Curacin

#### Origins

Curacin A, B, and C were isolated from the marine cyanobacterium *Lyngbya majuscule* from Curaçao (Caribbean) (Gerwick et al., 1994; Yoo and Gerwick, 1995), whereas curacin D was obtained from marine cyanobacterium *Lyngbya majuscule* from Virgin islands (Márquez et al., 1998).

#### Isolation, Purification, and Detection Methods

Curacin A was isolated through bioassay-guided partition of extract from *L. majuscule*. Preliminary, the collection of cyanobacteria from Caribbean were screened for the cytotoxic activity against Vero cell line (ATCC CCL81). Further, brine shrimp toxicity assay was performed to obtain the active fractions. The crude extract obtained in CHCl_3_/MeOH (2:1) from *L. majuscule* was processed through VC to give curacin A along with fatty acid contaminants. After subsequent analysis by NMR, GC-MS, and brine-shrimp toxicity, the bioactive component unreactive to CH_2_N_2_ was methylated and further purified by HPLC. Further analysis was performed by high resolution FAB-MS and 2D-NMR analysis (Gerwick et al., 1994). Subsequently, the exploration of minor metabolites related to curacin A led to isolation of two new structures named as Curacin B and Curacin C. The concentrated extract in CHCl_3_/MeOH (2:1) from *L. majuscule* were chromatographed over silica gel chromatography to obtain mixture of curacin A, B and C, with other contaminants. The curacin containing fraction was subjected to methylation (CH_2_N_2_) and acetylation (Ac_2_O/pyridine) to remove contaminants, and subsequently purified by normal-phase HPLC to obtain curacin B and C (Yoo and Gerwick, 1995).

Curacin D was isolated based on brine shrimp toxicity assay. The total extract obtained through multiple extraction of *L. majuscule* using CHCl_3_/MeOH (2:1), was subjected to silica gel vacuum chromatography to obtain step-wise gradient from 100% hexanes to 100% EtOAc. The fraction with toxicity to brine shrimp was further fractionated by RP-8 silica gel CC and final purification was performed with HPLC using 2 × 4.1 mm × 30 cm Versapack Silica 10 μm and 10% EtOAc in hexanes (Márquez et al., 1998).

#### General Chemical Structures

Curacins are mixed PKS/NRPS compound with unusual structural features. Curacin A shows unique chemistry containing cyclopropane group, thiazoline moiety, cis-alkenyl group, and terminal double bond (Gerwick et al., 1994). Curacin B and C are the geometrical isomer of curacin A, with difference of stereochemistry in the C-7 to C-10 diene and C-17 methyl and C-11 methylene groups. The geometry of C7 to C10 diene is 7(E),9(E) in case of curacin A and B, whereas curacin C has 7(E),9(Z) isomerism (Yoo and Gerwick, 1995). Curacin D also possess similar structure to Curacin A, while it lacks C17 methyl group, while retaining the geometry of C7 to C10 diene as 7(E),9(E) (Márquez et al., 1998). The chemical structure of curacin A–D are shown in Figure 2H.

#### Biological Activities

Curacin A was examined against various NCI cell lines and the cytotoxity pattern was evaluated. Subsequently, the inhibition of polymerization of tubulin was tested at different concentration of curacin A (2, 4, and 6 μM), which confirmed its cytotoxic effects. Further evaluation of effect of curacin A on growth of L1210 leukemia cells and Ca46 Burkitt lymphoma cells showed IC_50_ in the range of 9 × 10^−3^ and 2 × 10^−1^ μM, respectively. Similarly, the inhibition of ligand binding of curacin (10 μM) to tubulin in presence of colchicine and curacin (100 μM) was 76% and 4.6% respectively. Hence, it was confirmed that curacin A belongs to colchicine class of inhibitors (Gerwick et al., 1994). Similarly, the microtubule polymerization inhibition activity of mixture of curacin B and curacin C (95;5) was evaluated in 60 *in-vitro* cell line assay, whereas the mixture was less active than curacin A with IC_50_ value 1.6 μM and less potent inhibition of colchicine binding (65% at 5.0 μM) (Yoo and Gerwick, 1995). Curacin D also showed lower inhibition of colchicine binding (53% at 5.0 μM) and 7 fold lesser inhibition of tubulin polymerization (IC_50_ value ~4.8 μM) (Márquez et al., 1998). Due to its potential therapeutic potential as antimitotic agent, curacin A was evaluated for clinical trials (Simmons et al., 2005).

Further information on other secondary metabolites from *Lyngbya* sp. and their bioactivities are described in Table 2 and Figure 2.

**Table 2:** Lists of compounds from *Lyngbya* and biological activities.

**No**	**Chemical names**	**Cyanobacterial strains**	**Description of bioactivities**	**References**
1	Guineamides A-F	*L. majuscula*	Moderate cytotoxicity (a mouse neuroblastoma cell line with IC_50_ values 15 and 16 μM for guineamides B and C, respectively)	Tan et al., 2003
2	Ulongamides A-F	*Lyngbya* sp.	Weak cytotoxicity (KB cells)	Luesch et al., 2002a
3	Jahanyne	*Lyngbya* sp.	Inhibited the growth of human cancer cells (HeLa and HL-60 cells, with IC_50_ values 1.8 and 0.63 μM, respectively) and induced apoptosis in HeLa cells	Iwasaki et al., 2015b
4	15-norlyngbyapeptin A and lyngbyabellin D	*Lyngbya* sp.	Cytotoxicity (KB cell line with IC_50_ value 0.1 μM)	Williams et al., 2003
5	Malyngamide 3, cocosamides A and B	*L. majuscula*	Weak cytotoxicity (MCF7 and HT-29 cancer cells with IC_50_ values 29 and 48 μM, respectively)	Gunasekera et al., 2011
6	Lyngbouilloside	*L. bouillonii*	Modest cytotoxicity (neuroblastoma cells with IC_50_ value 17 μM)	Tan et al., 2002
7	Pitiprolamide	*L. majuscula*	Weak cytotoxicity (HCT116 and MCF-7 cancer cell lines with IC_50_ 33 μM for both) and anti-bacterial (*M. tuberculosis* and *B. cereus* with IC_50_ value 70 μM) activities	Montaser et al., 2011a
8	Lyngbyaureidamides A-B, Lyngbyazothrins C-D	*Lyngbya* sp.	Inhibit the 20S proteasome with IC_50_ values 7.1 and 19.2 μM, for lyngbyazothrins C and D, respectively	Zi et al., 2012
9	Malyngolide dimer	*L. majuscula*	*in-vitro* moderate anti-malarial activity (chloroquine resistant *Plasmodium falciparum* with IC_50_ 19 μM) but roughly equivalent toxicity against H-460 human lung cell lines	Gutierrez et al., 2010
10	Kalkipyrone	*L. majuscula* and *Tolypothrix* sp.	Toxic to brine shrimp (LD_50_ 3.01 μM) and gold fish (LD_50_ 6.02 μM)	Graber and Gerwick, 1998
11	Taisiamide F	*Lyngbya* sp.	Anti-proteolytic activity *in vitro* (cathepsins D and E, and BACE1 with IC_50_ values 57 × 10^−3^, 23 × 10^−3^, and 0.69 μM, respectively)	Al-Awadhi et al., 2016
12	Kurahyne	*Lyngbya* sp.	Anti-cancer (HeLa and HL-60 cells with IC_50_ values 3.9l and 1.5l M, respectively)	Iwasaki et al., 2015a
13	Kurahyne B	*Lyngbya* sp.	Anti-cancer (HeLa and HL-60 cells with IC_50_ values 8.1 and 9.0 μM, respectively)	Okamoto et al., 2015
14	Serinolamide A, propenediester	*L. majuscula* and *Oscillatoria* sp.	Moderate affinity for CB1 receptor (EC_50_ 2.3 μM)	Gutierrez et al., 2011
15	Desmethoxy-majusculamide C (DMMC)	*L. majuscula*	Potent anti-tumor activity with an IC_50_ value 20 nM against the HCT-116 human colon carcinoma	Simmons et al., 2005

### Microcystis

*Microcystis* are characterized by small cells organized into colonies. These colonies are bound by a thick mucilage composed of complex polysaccharide containing xylose, mannose, glucose, fucose, galactose, and rhamnose. They possess characteristics gas filled vesicles but lack individual sheaths. *Microcystis aeruginosa* is one of the harmful cyanobacteria because it is responsible for production of neurotoxins and hepatotoxins, such as microcystin and cyanopeptolin. These microorganisms along with their compounds causes formation of surface water bloom and leads to the mortality of the aquatic organism (Lee, 2018).

### Micropeptin

#### Origins

Different structures belonging to micropeptins, such as KB928, KB956, KB970A, KB970B, KB984, KB970C, KB1048, KB992, KB1046, TR1058, KR1030, KR1002, KR998, MZ845, MZ859, MZ939A, MZ925, MZ939B, MZ1019, MZ771, LH920, LH1021, LH1048, LH1062, LH911A, LH911B, LH911C, and LH925 were isolated from a *Microcystis* collected in diverse water reservoirs (fishpond, secondary water pond, etc.,) (Zafrir and Carmeli, 2010; Bladt et al., 2014; Elkobi-Peer and Carmeli, 2015; Hasan-Amer and Carmeli, 2017).

#### Isolation, Purification, and Detection Methods

The MeOH/H_2_O extract was fractionated by flash chromatography with RP-C18 open column, then combined with a bioassay-guided procedure to screen for bioactive fractions. Finally, the active fractions were subjected to size exclusion chromatography, and HPLC to obtain different variants of KB and MZ (Zafrir and Carmeli, 2010; Elkobi-Peer and Carmeli, 2015).

For other compounds, MeOH/H_2_O extract was separated by reversed phase (ODS) open column followed by separation on Sephadex LH-20. Further they were separated by preparative HPLC to yield different variants of KR and LH (Vegman and Carmeli, 2013; Bladt et al., 2014).

#### General Chemical Structures

**Figure 3:**
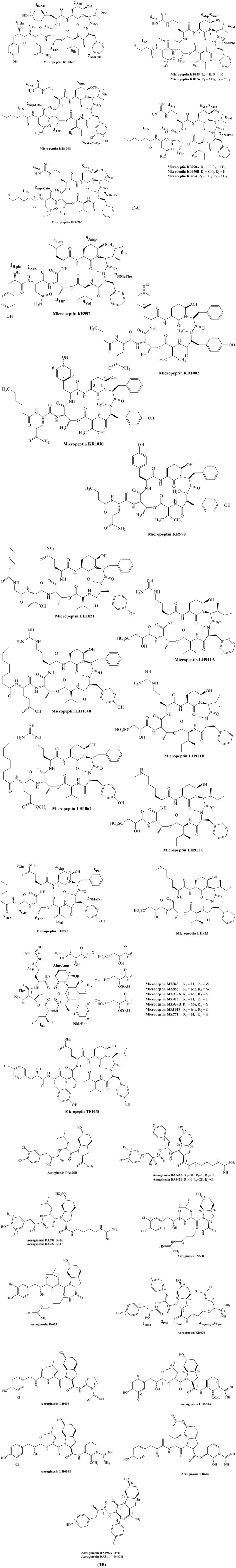
Chemical structures of bioactive molecules from *Microcystis*. **(A)** Micropeptins, **(B)** Aeruginosins.

Micropeptins are cyclic depsipeptides containing a skeleton peptide with a ring of six amino acids and a side chain of one to four amino acid units. The 19-membered core structure contains a unique 3-amino-6-hydroxypiperidinone (Ahp) moiety and an α-amino- β-hydroxy acid (L-threonine or β-hydroxy-γ-methylproline). The structure of micropeptin KR1002 contain the sequences of acid residues as Val, *N-*MeTyr, *N, N*-disubstituted-Phe, Ahp, HcAla, Thr, Gln, and BA (Bladt et al., 2014). Structure of several micropeptin analogs are depicted in Figure 3A.

#### Biological Activities

Micropeptins are protease inhibitor belonging to serine proteases. Several such variants such as KB928, KB956, KB970A, KB970B, KB984, KB970C, and KB1048 inhibited trypsin with sub-micromolar IC_50_. The micropeptins KB992 and KB1046 also inhibited chymotrypsin in a similar range of IC_50_ (Elkobi-Peer and Carmeli, 2015). Likewise, it was reported that micropeptin TR1058 inhibited chymotrypsin with an IC_50_ 6.78 μM (Hasan-Amer and Carmeli, 2017).

Bladt et al. (2014) evaluated the enzyme inhibitory activity of micropeptin KR1030, KR1002, and KR998 against serine proteases like chymotrypsin, elastase, and trypsin. It was concluded that IC_50_ of variants KR1030, KR1002, and KR998 against chymotrypsin was 13.9, 18.8 and 5.9 μM. The enzyme inhibitory activities of pure micropeptin MZ845, MZ859, MZ939A, MZ925, MZ939B, MZ1019, and MZ771 against serine proteases: trypsin, thrombin, chymotrypsin, and elastase were also determined (Zafrir and Carmeli, 2010). Micropeptins such as LH920, LH1021, LH1048, and LH1062 were responsible for the inhibition of chymotrypsin, while micropeptins LH1048, LH1062, LH911A, LH911B, LH911C, and LH925 were responsible for the inhibition of trypsin (Vegman and Carmeli, 2013).

### Aeruginosin

#### Origins

Aeruginosins IN608 and IN652 were isolated from *Microcystis* sp. strain BHU006, collected from Durgakund water reservoir in Varanasi, India (Elkobi-Peer et al., 2013).

Aeruginosins DA495A, DA511, DA642A, DA642B, DA688, DA722, DA495B, LH650A, LH650B, LH606, KB676, and TR642 were isolated in various locations in Israel (Adiv and Carmeli, 2013; Vegman and Carmeli, 2014; Elkobi-Peer and Carmeli, 2015; Hasan-Amer and Carmeli, 2017).

#### Isolation, Purification, and Detection Methods

The solvent extract from *Microcystis* sp. was fractionated using ODS flash column and Sephadex LH-20 column by eluting with a different type of solvent to obtain different fractions. Then they were subjected into RP-HPLC to purify the target compounds. For instance, the ODS flash column was eluted with a mixture of MeOH in water (3:7 MeOH/H_2_O to 9:1 MeOH/H_2_O) to obtain different fractions. Six bioassay-guided fractions showing inhibitory activities against trypsin and chymotrypsin were selected for Sephadex LH-20 column and eluted with 1:1 chloroform/methanol. Finally, the selected fractions were subjected to prep-HPLC using the gradient of MeOH and water with 0.1% trifluoroacetic acid to yield aeruginosin LH650A, LH650B, and LH606 (Vegman and Carmeli, 2014).

#### General Chemical Structures

Aeruginosin are class of short and linear peptides characterized by the presence of a hydroxyphenyl lactic acid (Hpla) derivative at the *N*-terminus of the peptide (first unit), a variable amino acid (Leu, Ile, Phe, or Tyr) (second unit), a 2-carboxy-6-hydroxyoctahydroindole (Choi) derivative (third unit), and an arginine derivative (if any) at the *C*-terminus of the peptide (fourth unit). The diversity in the structure is determined by the variability in each of the 4 acid units of their skeleton. For example, aeruginosins IN608 and IN652 contain four linear modified peptides as building blocks. Their structure contain sequence of amino-acids as D-*O*-Cl-Hpla-D-Leu-L-Choi-agmatine and D-*O*-BrHpla-D-Leu-L-Choi-agmatine, respectively (Elkobi-Peer et al., 2013). Similarly, the structure sequence of DA511, DA642A, and DA642B are D-Hpla-D-Tyr-L-6-*epi*-Choi-amide, L- *O*-Cl-Hpla-L-Phe-L-Choi-agmatine, and D- *O*-Cl-Hpla-L-Phe-L-Choi-agmatine, respectively (Adiv and Carmeli, 2013). The major structures of aeruginosin and its derivatives are shown in Figure 3B.

#### Biological Activities

Aeruginosins IN608 and IN652 inhibited trypsin with an IC_50_ value 4.3 and 4.1 μM, respectively (Elkobi-Peer et al., 2013). Aeruginosin DA642A showed inhibitory activity against trypsin and thrombin with IC_50_ value 30.8 and 15.5 μM, respectively. Aeruginosin DA642B, DA688, and DA722 inhibited trypsin but not thrombin. However, both aeruginosins DA495A and DA511 did not inhibit trypsin and thrombin (Adiv and Carmeli, 2013). Aeruginosins LH650A, LH650B, and LH606 showed mild inhibition against trypsin whereas higher inhibition against thrombin (Vegman and Carmeli, 2014). Aeruginosin KB676 inhibited trypsin with sub-μM IC_50_ but aeruginosin TR642 inhibited trypsin and thrombin (Elkobi-Peer and Carmeli, 2015; Hasan-Amer and Carmeli, 2017).

Other bioactive compounds from microcystis are described in Table 3 and Figure 3.

**Table 3:** Lists of compounds from *Microcystis* and biological activities.

**No**	**Chemical names**	**Cyanobacterial strains**	**Description of bioactivities**	**References**
1	Microcystbiopterins A-E	*Microcystis* spp.	Their anti-microbial and cytotoxic properties were evaluated but all were found not active in both assays	Lifshits et al., 2016
2	Microcystins 1–4	*M. aeruginosa*	Dose-dependent cytotoxicity (HCT-116 cells with estimated EC_50_ values 2.9, 3.3, and 11 μM, respectively; A549 cells with estimated EC_50_ values 0.45, 0.36, and 1.3 μM, respectively, for microcystins 1, 3, and 4)	He et al., 2018
3	Microcyclamide	*M. aeruginosa*	Moderate cytotoxicity (P388 murine leukemia cells with IC_50_ value 2.06 μM)	Ishida et al., 2000
4	Microvirin (MVN)	*M. aeruginosa*	Anti-HIV-1 activity (with IC_50_ values ranging between 2.1 × 10^−3^ and 0.167 μM).	Huskens et al., 2010
5	[D-Leu1] MC-LR	*M. aeruginosa*	Anti-mycobacterial (*M. tuberculosis* with MIC between 1.93 and 0.06 μM) and cytotoxicity (HTC cell line)	Ramos et al., 2015

### Classification of Bioactive Compounds

Generally, the secondary metabolites from *Nostoc, Lyngbya*, and *Microcystis* can be structurally classified into different classes such as peptide, polyketide, lipid, alkaloid, and others. But the major compounds belong to polyketides or peptides or hybrid of them. These interesting structural aspects are responsible for determining their biological activities. The lists of major chemical structures obtained from these important cyanobacterial genera are described in Tables 4, 5.

**Table 4:** Lists of polyketides and peptide class compounds.

**No**	**Cyanobacterial strains**	**Name of peptides**	**Biological activities**	**References**
1	*Nostoc* sp. CENA 219, *Nostoc calcicula* 6 sf Calc	Hassallidins	Anti-fungal	Shishido et al., 2015
2	*Nostoc* sp.	Hassallidin C–D	Anti-fungal	Vestola et al., 2014
3	*Nostoc* sp. GSV 224	Cryptophycins-46,−175, and−176	Cytotoxicity	Subbaraju et al., 1997
4	*Nostoc* sp. GSV 224	Cryptophycins-38,−326, and−327	Cytotoxicity	Chaganty et al., 2004
5	*Nostoc* sp. CENA543	Pseudospumigins A-F	Protease inhibitors	Jokela et al., 2017
6	*Nostoc* sp. ATCC53789	Nostocyclopeptides A1-A2	Weak cytotoxicity	Golakoti et al., 2001
7	*Nostoc* sp.	Cryptophycin 52	Anti-tumor activity	Shih and Teicher, 2001
8	*Nostoc* sp. HAN11/1	7-*O*-Me-scytophycin B	Potent antifungal	Shishido et al., 2015
9	*Nostoc* sp. UHCC 0450	Swinholides	potent cytotoxicity	Humisto et al., 2017
10	*L. majuscula*	Lagunamides A-B	Anti-malarial activity and cytotoxicity	Tripathi et al., 2010
11	*L. majuscula*	Trungapeptins A-C	Mild brine shrimp toxicity and mild ichthyotoxicity	Bunyajetpong et al., 2006
12	*L. majuscula*	Apratoxin A	Potent cytotoxicity *in vitro*	Luesch et al., 2001b
13	*Lyngbya* sp.	Apratoxins B-C	Cytotoxicity	Luesch et al., 2002c
14	*L. majuscule* and *L. sordida*	Apratoxin D	Potent cytotoxicity *in vitro*	Gutierrez et al., 2008
15	*L. bouillonii*	Apratoxin E	Strong cytotoxicity	Matthew et al., 2008
16	*L. bouillonii*	Apratoxins F and G	Potent cytotoxicity	Tidgewell et al., 2010
17	*L. majuscula*	Guineamides A-F	Moderate cytotoxicity	Tan et al., 2003
18	*L. majuscula*	Pitipeptolides A-B	Weak cytotoxicity, moderate anti-mycobacterial activity and stimulate elastase production	Luesch et al., 2001a
19	*L. majuscula*	Pitipeptolides C-F	Potent microbial activity	Montaser et al., 2011b
20	*Lyngbya* sp.	Ulongamides A-F	Weak cytotoxicity	Luesch et al., 2002a
21	*Lyngbya* sp.	Jahanyne	Cytotoxicity	Iwasaki et al., 2015b
22	*Lyngbya* sp.	Lyngbyabellin D	Cytotoxicity	Williams et al., 2003
23	*L. majuscula*	Cocosamides A-B	Weak cytotoxicity	Gunasekera et al., 2011
24	*L. majuscula*	Pitiprolamide	Weak anti-bacterial activity and cytotoxicity	Montaser et al., 2011a
25	*Lyngbya* sp.	Lyngbyazothrins C-D	Inhibit the 20S proteasome	Zi et al., 2012
26	*Lyngbya* sp.	Tesiamide F	Anti-proteolytic activity *in-vitro*	Al-Awadhi et al., 2016
27	*Lyngbya* sp.	Kurahyne B	Anti-cancer	Okamoto et al., 2015
28	*Lyngbya* sp.	Lyngbyaloside B	Weak cytotoxicity	Luesch et al., 2002b
29	*L. majuscula*	Malyngamide 3	Weak cytotoxicity	Gunasekera et al., 2011
30	*L. bouillonii*	Lyngbouilloside	Modest cytotoxicity	Tan et al., 2002
31	*L. majuscula*	Malyngolide dimer	Anti-malarial activity and cytotoxicity	Gutierrez et al., 2010
32	*Lyngbya* sp.	Kurahyne	Anti-cancer	Iwasaki et al., 2015a
33	*M. aeruginosa*	Microcyclamide	Moderate cytotoxicity	Ishida et al., 2000
34	*M. aeruginosa*	Microcystins 1–4	Dose-dependent cytotoxicity	He et al., 2018
35	*M. aeruginosa*	[D-Leu1] MC-LR	Anti-mycobacterial activity and cytotoxicity	Ramos et al., 2015

**Table 5:** List of other class compounds.

**No**	**Cyanobacterial strains**	**Name of other compounds**	**Biological activities**	**References**
1	*N. verrucosum*	(*9Z,12Z*)-9,12,15-hexadecatrienoic acid (n-1 fatty acid)	Anti-bacterial activity	Oku et al., 2014
2	*L. majuscula*	Serinolamide A	Moderate affinity for CB1 receptor	Gutierrez et al., 2011
3	*Nostoc* sp. (TAU strain IL-235)	Prenostodione	UV-absorbing activity	Ploutno and Carmeli, 2001
4	*Nostoc* sp. MGL001	EMTAHDCA	Anti-bacterial activity	Niveshika et al., 2016
5	*N. commune* Vauch	Reduced scytonemin	Anti-tumor activity	Itoh et al., 2014a
6	*N. commune* Vauch	Scytonemin	Ultraviolet-absorbing activity	Itoh et al., 2014a
7	*N. verrucosum*	(*9Z,12Z*)-9,12,15-hexadecatrienoic acid (*n*-1 fatty acid)	Anti-bacterial activity	Oku et al., 2014
8	*L. majuscula*	Serinolamide A	Moderate affinity for CB1 receptor	Gutierrez et al., 2011
9	*Nostoc* sp.	Carbamidocyclophanes F-G	Anti-bacterial activity and cytotoxicity	Luo et al., 2014
10	*Nostoc* sp. CAVN2	Carbamidocyclophanes H-L	Anti-bacterial activity and cytotoxicity	Preisitsch et al., 2015
11	*Nostoc* sp. CAVN2	Carbamidocyclophanes M-U	Anti-bacterial activity	Preisitsch et al., 2016
12	*N. flagelliforme*	Nostoflan	Anti-viral activity	Kanekiyo et al., 2005
13	*Nostoc* sp.	Cylindrocyclophanes A_4_-A_1_, C_4_-C_1_, F_4_, A_B4_	Inhibition of the 20S proteasome and cytotoxicity	Chlipala et al., 2010
14	*N. commune*	Comnostins A-E	Anti-bacterial activity and cytotoxicity	Jaki et al., 2000b
15	*N. commune* Vauch	Nostocionone and its derivatives	Strong anti-bacterial and anti-inflammation	Itoh et al., 2014b
16	*N. commune* Vauch	NVPS (polysaccharide from *Nostoc commune* Vauch)	Cytotoxicity	Guo et al., 2015
17	*Nostoc* sp. TAU strain IL-220	Nostocyclyne A	Anti-microbial activity	Ploutno and Carmeli, 2000
18	*N. commune*	A diterpenoid, an anthraquinone and an indane derivative	Anti-bacterial activity	Jaki et al., 2000a
19	*L. majuscula* and *Tolypothrix* sp.	Kalkipyrone	Toxic to brine shrimp and gold fish	Graber and Gerwick, 1998
20	*Microcystis* spp.	Microcystbiopterins A-E	No anti-microbial and cytotoxic	Lifshits et al., 2016
21	*M. aeruginosa*	Microvirin	Potent anti-viral activity	Huskens et al., 2010

## Multi-Omics Approaches for Uncovering Bioactive Compounds From Cyanobacteria

The secondary metabolites from cyanobacteria possess immense diversities and activities, hence there have been significant interest in the study of their structures, genetics/biosynthetic mechanisms, and bioactivities. But, there is a transition of research from traditional screening approaches focused on chemical structures to molecular engineering and strain engineering based on metabolic engineering and synthetic biology in recent times (Mazard et al., 2016). The availability of diverse –omics data and technologies based on such information has provided a great resource for discovery of the potential compounds and study of their biosynthetic pathways. For example, the comparative genomics approach was employed for uncovering the distinct metabolic potential of the cyanobacterial genera. For example, the complete genome sequence of *Lygnea* was obtained through a variety of computational tools and assembly methods. The comparative analysis revealed the discrepancy in their metabolic capability and biosynthetic potential, whereas diverse natural product biosynthetic gene cluster (BCG) were predicted (Leao et al., 2017). The genome of many cyanobacteria strains are already obtained, for e.g., *N. punctiforme* ATCC 29133 was sequenced which provided a better understanding about the distribution of secondary metabolites gene clusters (Moraes et al., 2017). Such availability of genome information can be combined with specific analytical tools to obtain the natural products and their biosynthetic pathways, which is most commonly defined as “genome mining” approach (Zerikly and Challis, 2009; Thuan et al., 2018). For example, the combination of mass spectrometric based metabolite profiling and genome analysis was established as a very powerful tool for obtaining columbamides, a new class of di- and trichlorinated acyl amides from cyanobacteria (Kleigrewe et al., 2015). Similarly, the analysis of genomic information in context to the presence of particular biosynthetic enzymes can be employed for revealing the unusual chemistries in diverse natural products produced by cyanobacteria (Coates et al., 2014). By a combination of matrix-assisted laser desorption ionization (MALDI) and isotope-labeling, the dynamics of biosynthesis of jamaicamide was studied, which even revealed the presence of a sub-milligram level of biosynthetic intermediates (Esquenazi et al., 2011). Similarly, the comparative analysis of the genome of *M. aeruginosa* NIES 298 and *M. aeruginosa* PCC 7806 in respect to microcyclamide-like gene cluster, and structural elucidation of metabolites by MS and NMR spectroscopy provided insight on the biosynthesis of microcyclamide in two strains of *M. aeruginosa* (Ziemert et al., 2008). Such inter-complementation studies whereas the biosynthetic studies are supported by the genomic information and metabolite profiling has revolutionized the study of bioactive compounds from cyanobacteria.

The transcriptional studies of cyanobacteria provide useful information about the expression mechanism and techniques for targeting their genetic manipulation. For example, the transcription analysis of jamaicamide gene cluster from the marine cyanobacterium *Lyngbya* majuscule revealed information about the expression level of each biosynthetic gene and regulatory protein involved in biosynthetic pathways (Jones et al., 2009).

## Heterologous Production of Cyanobacterial Compounds

Heterologous expression is an efficient approach for production or engineering of diverse natural products from cyanobacteria (Knoot et al., 2018). The barbamide biosynthetic gene cluster from a cyanobacterium *Moorea producens*, was heterologously expressed in *Streptomyces venezuelae* (a terrestrial actinobacteria), resulting in the production of a new barbamide congener 4-*O*-demethylbarbamide (Kim et al., 2012). Similarly, direct pathway cloning (DiPaC) (an BGC capturing strategy based on long-amplicon PCR and *in vitro* DNA assembly) was utilized for the heterologous production of anabaenopeptins from *Nostoc punctiforme* in *E. coli* BAP1 cells (Greunke et al., 2018). Similarly, heterologous expression of lyngbyatoxin BGC from an uncultured marine cyanobacterium *Moorea producens* was performed in *E. coli*, leading to a high-titer production of lyngbyatoxin A 12 (58.6 μM) and its precursor indolactam V (498.3 μM) (Ongley et al., 2013). Similarly, the heterologous expression of the lyngbyatoxin BGC using replicative plasmids led to the production of lyngbyatoxin A in *Anabaena* 7120 with yields comparable to the native producer (Videau et al., 2016).

The systematic modulation of metabolic pathways by fine-tuning the expression levels of proteins by engineering promoters, RBS (ribosome binding sites) or other regulators is an efficient approach for metabolic engineering of native or heterologous hosts (Dhakal and Sohng, 2017; Dhakal et al., 2017). A promoter library and aRBS library was constructed, and evaluated to check the efficiency of the expression level of heterologous genes in *Synechocystis* sp. PCC 6803. This study presented an efficient genetic toolbox that can be used to fine-tune gene expression levels and moderate metabolic bottlenecks in cyanobacteria (Wang et al., 2018). Inducible promoters allowing flexible control over gene expression are very effective tool for tuning the expression levels. So a rhamnose-inducible rhaBAD promoter of *E. coli* was introduced into *Synechocystis* sp. PCC 6803 (a model freshwater cyanobacterium). It was demonstrated that it had superior properties compared to previous reports of inducible promoter systems in cyanobacteria (Kelly et al., 2018). The native promoter from *M. aeruginosa* PCC 7806(native producer) was replaced for the heterologous production of microcystin-LR and [D-Asp3] microcystin-LR from *E. coli* GB05-MtaA (Cullen et al., 2018). Similarly, the recombinant microcystin synthetases gene cluster from *M. aeruginosa* PCC 7806 was heterologously expressed in *E. coli* to yield significant titer of [d-Asp3] microcystin-LR and microcystin-LR. This gene cluster was assembled by Red/ET recombineering, and the native promoters were also replaced with an inducible PtetO promoter to obtain titer of microcystin superior to *M. aeruginosa* (Liu et al., 2017).

## Conclusions and Future Trends

Important members of genera *Cyanobacteri*a as *Nostoc, Lyngbya*, and *Microcystis* are distributed worldwide in various environmental locations. The evolution of those bacteria over a long duration through adaption to diverse environmental conditions must have lead to the capability for the biosynthesis of diverse types of bioactive compounds to support their survival and competition with other organisms. Until now, numerous secondary metabolites have been discovered, purified and structurally characterized by using advancement in isolation strategies and precise separation with diverse chromatographic methods. Subsequently, these compounds were evaluated for anti-fungal, anti-bacterial, antiviral and antiproliferative activities, etc., and thus established as new pharmaceutical lead compounds based on diverse bioactivity assays. The present and future trends in the research of these genera include integrated multi-omics analysis for detection of biosynthetic gene clusters, biosynthetic pathways, and metabolic engineering for enhanced production of target compounds. The metabolomic analysis of field-collected materials by using the tool as LC-TOF-ESI-MS/MS, HR-ESI-MS, MALDI-TOF-MS, etc., are assisting the dereplication of the known compounds, and search for novel molecules. In addition, the development of advanced culture techniques by mimicking the natural conditions have immense potential to uncover novel molecules from previously un-isolatable or un-cultivable strains. The metagenomics approach, on the other hand, is facilitating the culture-independent isolation of biosynthetic pathways. Most importantly the application of advanced molecular biology method and synthetic biology tools such as CRISPER-Cas9 or synthesis of artificial genetic parts/large genomic segments have elaborated the metabolic engineering of cyanobacteria to the next level. In addition, the biomimetic chemical synthesis is complementing the biological engineering for biosynthesis or chemo-biosynthesis of novel derivatives of compounds from cyanobacteria.

## Author Contributions

NT, TA, DD, NC, AS, and JS conceived the review. NT and DD wrote the draft of the manuscript. All authors reviewed the manuscript.

### Conflict of Interest Statement

The authors declare that the research was conducted in the absence of any commercial or financial relationships that could be construed as a potential conflict of interest.
